# Reducing rates of *Clostridium difficile* infection by switching to a stand-alone NAAT with clear sampling criteria

**DOI:** 10.1186/s13756-018-0332-2

**Published:** 2018-03-12

**Authors:** E. Casari, C. De Luca, M. Calabrò, C. Scuderi, C. Daleno, A. Ferrario

**Affiliations:** Microbiology Unit, Analysis Laboratory, Humanitas Research Hospital, via Manzoni, 56 20089 Rozzano, Milan, Italy

**Keywords:** *Clostridium difficile*, NAAT test, Hospital CDI, Prevention

## Abstract

**Background:**

*Clostridium difficile* infection is an important cause of morbidity and mortality but the optimal method of diagnosis for both patient management and infection prevention remains controversial.

**Methods:**

Our hospital made a decision to switch from the use of toxin immunoassay to a stand-alone nucleic acid test. This change was accompanied by the provision of clear sampling guidance and rejection criteria and this study aimed to assess the impact of that change. We analysed sample numbers, numbers of positive results, and the proportion of cases assessed as healthcare acquired over a 6-year period during which the testing method was changed from a toxin A/B immunoassay to a stand-alone commercial nucleic acid test after the first two years.

**Results:**

Sample numbers and numbers of cases assessed as healthcare acquired fell following the introduction of the nucleic acid test and sampling guidance, while infection rates in other hospitals in the same region remained relatively stable.

**Conclusions:**

It is our opinion that the use of a highly sensitive assay together with clear sampling guidance offers the optimal approach to patient management and best use of isolation facilities.

## Introduction

*Clostridium difficile* infection (CDI) continues to cause significant morbidity, mortality, and increased hospital length of stay [1] but the optimal method of diagnosis remains controversial. Rapid assays to detect *C difficile* toxins A and B by enzyme immunoassay are reported to have poor sensitivity and their performance varies markedly across manufacturers [2]. Glutamate dehydrogenase (GDH) immunoassay may be a useful assay for screening out patients who do not carry *C difficile* but does not differentiate toxigenic strains and has been shown to fail to detect approximately 10 to 15% of patients with CDI [3]. Nucleic acid amplification tests (NAATs) detecting *tcdB* genes are highly sensitive for toxigenic *Cdifficile* strains, and assays such as the Cepheid Xpert*C difficile* PCR test provide results in under an hour and can be performed on-demand, however it has been suggested that they may overcall the diagnosis of CDI in some cases and acquisition costs are higher than EIAs. Although routes of transmission of *C difficile* are not always entirely clear, prevention of transmission within healthcare settings remains a priority, and the key means by which this is achieved is through prompt isolation of patients with diarrheal symptoms compatible with CDI and rapid testing to identify those with the condition. A great many patients in hospitals have symptoms of diarrhoea but in up to 90% of cases this has a non-infectious cause [4]. Many hospitals lack sufficient facilities and resources to isolate all patients with loose stools, so a sensitive and rapid assay is a priority to exclude those patients who do not require isolation and prioritise resources for those who do. Despite this, economic factors do influence decisions regarding testing, so the impact of different assays is also important in the decision making regarding the optimal sampling and testing strategy.

In 2012, after many years of using toxin immunoassays, our hospital decided to introduce a commercial NAAT as a stand-alone test for the diagnosis of CDI, with clear guidance and rejection criteria for submission of samples. This retrospective analysis shows the impact of this strategy, based on rapid results, greater sensitivity of the assay, and shortened time to initiation of isolation of positive cases, on CDI rates.

## Methods

This was a single centre retrospective analysis of *C difficile* sample numbers and numbers of infections over a 6-year period (January 2010 to December 2015) during which the testing method was changed from toxin A and B enzyme immunoassay (EIA) to a *C difficile* NAAT assay. The study centre is a 750-bed tertiary care university hospital in the south of Milan characterized by high risk populations, of our admissions more than an half are for surgery, with a Bone Marrow Transplant Unit and Cancer Unit, and a total of 25 ICU beds.

During the first 2 years, all samples were tested with a toxin A/B enzyme immunoassay (TOXA/B QUIK CHEK, Techlab, Blaksburg, VA) and in the following four years testing was by a commercial NAAT assay (Xpert*C difficile*, Cepheid, Sunnyvale, CA) as a stand-alone test. All tests were performed strictly in accordance with manufacturer’s instructions.

During the period when the EIA toxin test was used there were no clear guidelines for sampling. Following the introduction of the NAAT clear criteria for testing were implemented and a detailed internal guideline was emailed to all clinicians explaining that only unformed stool samples were to be tested and in the case of a previous positive result the patient would only be re-tested after 30 days and in the case of a previous negative result only after 10 days. Samples not meeting these criteria were rejected and the microbiology laboratory did not examine the sample explaining the reason in the laboratory report. Clinicians could over-rule these decisions in discussion with infection specialists in cases where special circumstances pertained.

Positive cases were reported by telephone to the physician or ward nurse immediately they became available and this was followed up by an email to the Infection Prevention team. Positive patients who were not already in a single room were placed in isolation or, where this was not possible, cohorted in a double room with another *C difficile* positive patient. Positive patients remained in isolation until resolution of symptoms. Following patient discharge strict environmental cleaning and disinfection of the isolation room was carried out.

Study patients included all those for whom CDI was suspected and one or more samples submitted for a CDI diagnostic test. Each case of CDI was reviewed by an epidemiology nurse and evaluated as to whether healthcare or community acquired. The definition of healthcare acquired used in this study was the appearance of symptoms more than 48 h following admission to hospital.

## Results

**Fig. 1:**
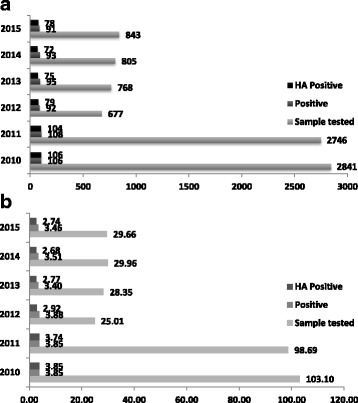
Number of sample tested, positive sample and those categorised as healthcare acquired from 2010 to 2015 (**a**) and in terms of rates per 1000 admissions (**b**)

A total of 8680 samples were tested for CDI over the study period – 2841, 2746, 677, 768, 805, and 843 tests in 2010, 2011, 2012, 2013, 2014 and 2015 (Fig. 1a). During 2012 only 46 EIA tests were performed through the transition period. For the corresponding years, the total number of positive samples and those categorised as healthcare acquired was 106/105 for 2010, 108/104 for 2011, 92/79 for 2012, 95/75 for 2013, 93/76 for 2014 and 91/78 for 2015 respectively (Fig. 1a). In order to compare the data during the whole period of the study and to minimize the differences in inpatient time and number of patients they have been expressed in terms of rates per 1000 admissions (Fig. 1b).

**Fig. 2:**
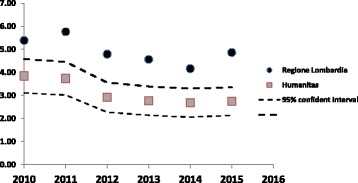
Number of positive cases /1000 admissions of our hospital compared with the number of positive cases of the other hospital in Lombardia region

To put these results and the reductions in positive cases in context, our results, expressed in terms of rates per 1000 admissions, have been compared with similar results in other Lombardia region hospitals as reported to the regional epidemiology observatory (https://logindwh.servizirl.it/erogatore-servizio/accessoportali/indexPortal.jsp) obtaining a statistical significance difference (*p* < 0,05) (Fig. 2).

## Discussion

This study showed that moving from a toxin EIA to a stand-alone NAAT resulted in fewer samples tested and lower positivity rates, largely due to a reduction in the number of healthcare associated cases. The reasons for these findings are likely to be multifactorial. Lack of confidence in the sensitivity of the toxin tests meant that clinicians often repeated the test up to three or more times before declaring the patients free from *C difficile* infection and releasing them from isolation, resulting in a poor use of isolation facilities. One limitation of our study, however, is that we have been unable to quantify the number of repeat samples in the period before the introduction of the NAAT.

Our decision to use a stand-alone test rather than an algorithmic approach meant that results were available in a timely manner with no requirements for batching of samples, thus reducing the time from initiation of isolation to receipt of a result to the shortest possible time and maximizing the use of isolation facilities. Our experience of decreasing incidence of healthcare acquired CDI contrasts with a relatively steady state in other hospitals in the same region, suggesting that our testing strategy was likely to have had an impact on our findings. Isolation of cases and decontamination of the environment appear to be the most important factors in preventing transmission. In this study protocols for decontamination of the environment remained unchanged throughout the study period. Similarly, antimicrobial stewardship remains a critical factor in the prevention of CDI, however our hospital has had an active and unchanged antimicrobial stewardship programme in place since 2010. Our aim was to provide an assay that was both sufficiently sensitive to detect carriers of *C difficile* and timely enough to allow for best use of isolation facilities. Other studies [5] have shown an impact of using NAAT testing combined with improved infection prevention and decontamination strategies, and a recent study using whole genome sequencing [6] has confirmed that symptomatic patients who harbor toxigenic strains of *C difficile* contribute to transmission even when they are faecal toxin negative.

The best testing strategy to encompass management of individual patients and prevention of transmission remains controversial. Most guidelines from professional societies no longer recommend toxin immunoassays as stand-alone tests and some European guidelines suggest strategies involving an algorithmic approach with two or even three stage testing [7–9]. These can cause confusion with interpretation for clinicians and may introduce delays in initiating management of patients because of the longer time to results, especially where batching of some assays is required. On the other hand some studies [10, 11] suggest that there may be over- diagnosis of *Clostridium difficile* infection in the molecular test era and that the presence of pre-formed toxin is a better marker of symptomatic infection. In our setting we feel that the important role of infectious disease physicians in reviewing cases and deciding on management based on clinical findings has helped to mitigate the possibility of inappropriate treatment based on laboratory results alone. Moreover some recent reports [12, 13] suggesting that the CT value of some commercial NAATs may predict the presence of pre-formed toxin and even more severe disease offers an interesting possibility of using this assay to further guide management.

In summary, our findings suggest that the use of a rapid and sensitive commercial NAAT as a stand-alone assay together with clear sampling guidance offers the optimal approach to patient management and best use of isolation facilities.
